# Intracellular pathogens under attack

**DOI:** 10.7554/eLife.14729

**Published:** 2016-02-19

**Authors:** Petr Broz

**Affiliations:** Focal Area Infection Biology, Biozentrum, University of Basel, Basel, Switzerlandpetr.broz@unibas.ch

**Keywords:** guanylate-binding proteins, host-pathogen interaction, cell-autonomous immunity, multiparameter fluorescence image spectroscopy, Förster resonance energy transfer, immune response, Mouse

## Abstract

Antimicrobial proteins deliver a double punch that can destroy the *Toxoplasma gondii* parasite and its niche inside cells.

**Related research article** Kravets E, Degrandi D, Ma Q, Peulen TO, Klumpers V, Felekyan S, Kuhnemuth R, Weidtkamp-Peters S, Seidel CA, Pfeffer K. 2016. Guanylate binding proteins (GBPs) directly attack *T. gondii* via supramolecular complexes. *eLife* **5**:e11479. doi: 10.7554/eLife.11479

**Image** Fluorescence-based techniques were used to study guanylate-binding proteins in cells infected with the *T. gondii* parasite

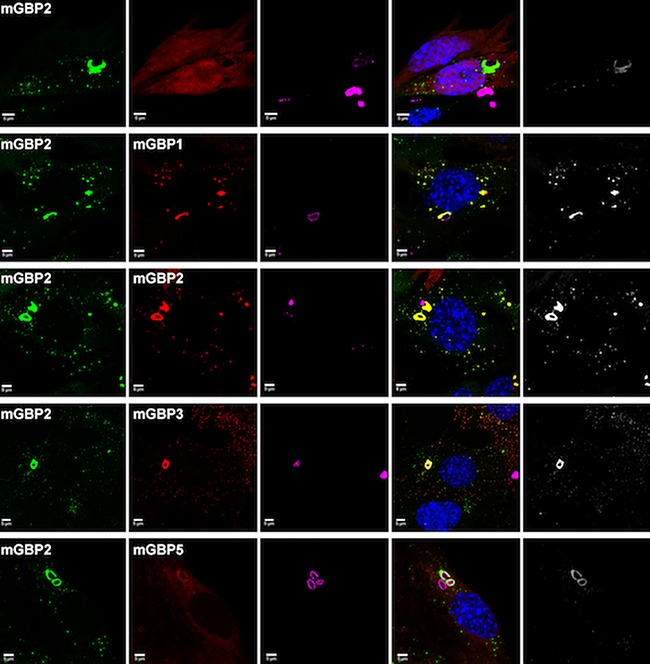

The immune system provides a formidable barrier that prevents us from being overwhelmed by the numerous microbial pathogens that we encounter every day. However, many pathogens manage to escape the immune system by invading cells and replicating within them. To counter these pathogens immune cells produce a powerful cytokine called interferon-γ that reprograms the invaded cells to express hundreds of antimicrobial proteins. These proteins transform the cells into a hostile environment for pathogens.

Among the most abundant of the antimicrobial proteins is a group of enzymes that bind and hydrolyze guanosine triphosphate (GTP). Nucleotide binding allows these enzymes, which are called guanylate-binding proteins (GBPs), to form oligomers (Kravets et al., 2012; Praefcke et al., 2004). Several GBPs also contain hydrophobic groups that enable them to associate with membranes. GBPs are highly conserved in vertebrates and essential for the cell-autonomous defense in vivo against many intracellular pathogens.

Mice deficient in GBPs are highly susceptible to infections with a protozoan parasite called *Toxoplasma gondii* that is commonly found in the developed world, and which can cause serious and often fatal infections in people with weakened immune systems (Yamamoto et al., 2012). *T. gondii* replicates within host cells in a membranous compartment known as the parasitophorous vacuole (PV). Intriguingly the antimicrobial function of GBPs is tightly linked with their ability to target and disturb the membrane of this vacuole (Degrandi et al., 2007; Degrandi et al., 2013; Selleck et al., 2013). However, relatively little is known about the ways in which proteins co-operate to restrict pathogen replication.

Now, in eLife, Klaus Pfeffer, Claus Seidel and co-workers at the Heinrich-Heine University Düsseldorf – including Elisabeth Kravets, Daniel Degrandi and Qijun Ma as joint first authors – report a comprehensive analysis of how different GBPs assemble into large complexes to control the replication of *T. gondii* (Kravets et al., 2016). They also demonstrate for the first time that in addition to targeting the PV, the GBPs attack the parasite directly.

Kravets et al. used a technique called multi-parameter fluorescence imaging spectroscopy (MFIS) to study fluorescently-tagged murine GBP2 in real-time. The data show the pre-assembly of dimers and oligomers containing mainly GBP2, but also GBP1 and GBP3, in vesicle-like structures in the cytosol of uninfected cells (Figure 1). Following infection the GBPs rapidly moved to the vacuole containing *T. gondii*, where they formed even larger multimers. Fluorescence measurements revealed the staggering size of these multimers: they contained between 1000 and 6000 monomer units and measured several hundred nanometers across. The ability of GBP2 to form multimers at the vacuole was absolutely essential for its immune function: GBP2 mutants that lacked the ability to bind and hydrolyze nucleotides, or to associate with membranes, failed to control the replication of *T. gondii*.

**Figure 1.**
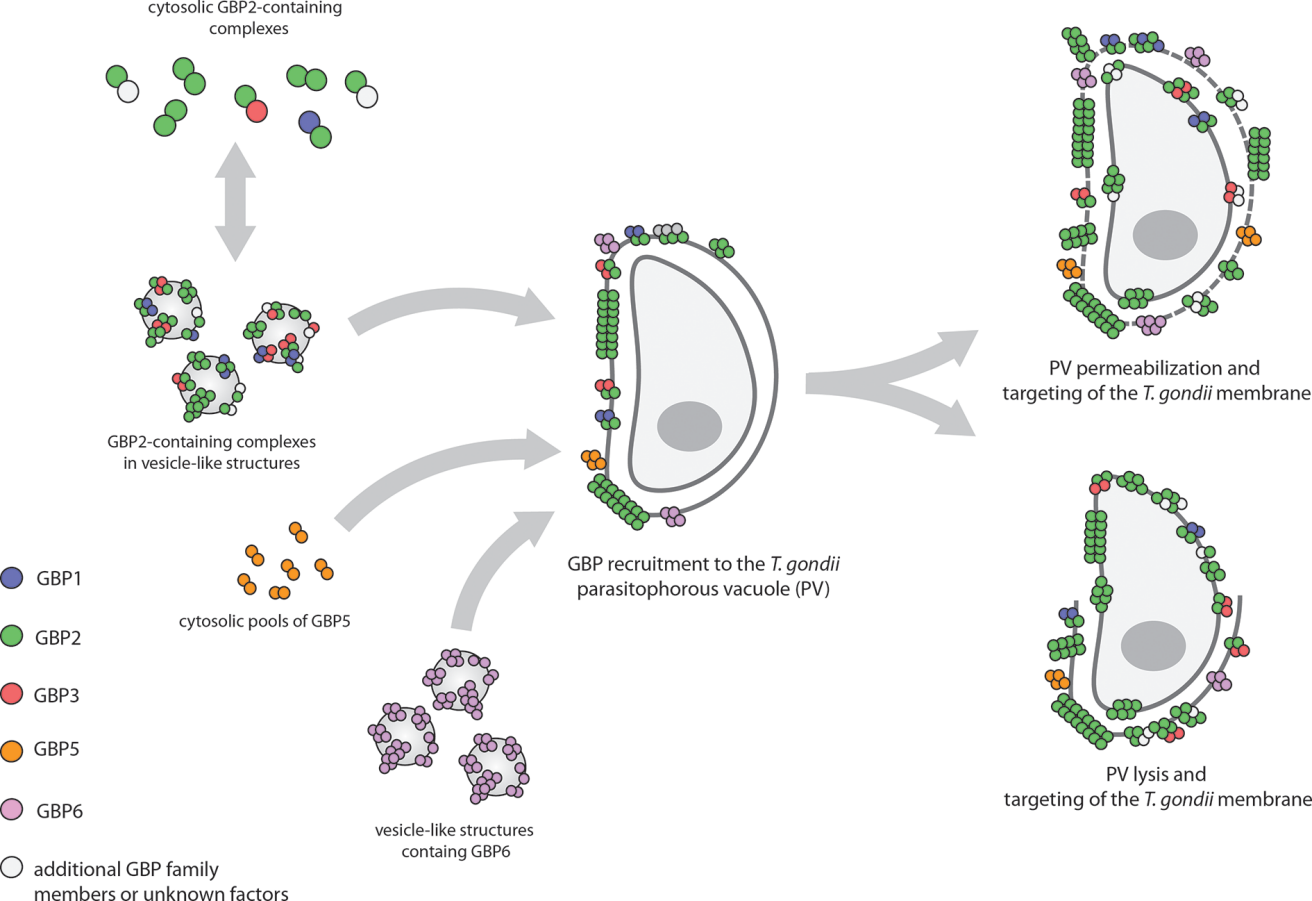
Guanylate-binding proteins (GBPs) and the parasite *T. gondii*. A number of different GBPs are involved in targeting *T. gondii*. GBP2 forms homodimers and heterodimers with GBP1 and GBP3 (and possibly other GBP family members) in the cytosol, or small oligomers in vesicle-like structures (top left). GBP5 forms homodimers in the cytosol, while GBP6 forms small oligomers in vesicle-like structures (bottom left). When *T. gondii* infects the cell, these GBPs move to the membrane of the parasitophorous vacuole (PV) inside which the parasite will replicate, and assemble into large multimers. The GBPs then either permeabilize the PV and accumulate on the parasite (top right), or completely disrupt the PV and target the membrane of the parasite (bottom right).

But how do the multimers restrict parasite growth? Live cell imaging revealed that after targeting the PV, the GBPs either penetrated or lyzed the membrane of the vacuole, and then went on to target the plasma membrane of the parasite itself. Although the fate of the parasites after this attack was not examined in detail, Kravets et al. note that they must have been killed because they displayed signs of membrane permeabilization.

The work of Kravets et al. is a landmark in the field because it firmly establishes that GBPs are part of a novel intracellular immune system that has the ability to recognize specific intracellular pathogens and to attack both the parasite itself and the vacuolar compartment in which it replicates. However, the work also raises a number of important questions. For example, we still do not know how GBPs specifically target the PV and distinguish the cell's own membranes from other membranes. Intriguingly, a group of related proteins, the immunity-related GTPases, are also known to destabilize the PV (Zhao et al., 2009), which might require cooperation with GBPs (Haldar et al., 2015; Yamamoto et al., 2012). It is thought that both groups of proteins rely on components of the autophagy system to target the PV, but the exact cues that are recognized on the PV membrane have not been identified yet.

Furthermore, what is the structure and composition of the large multimers formed by the GBPs, and how do they destabilize the membranes of the PV and the pathogen? It is notable that GBPs are related to the dynamin superfamily of GTPases, which has a central role in endocytosis (Ferguson and De Camilli, 2012). This process is relatively well understood: it starts with the Dynamin forming a helical oligomer around the neck of a nascent vesicle; GTP hydrolysis then leads to the helix being extended lengthwise and becoming narrower; eventually the helix breaks and the vesicle is 'pinched off' from the membrane. The large multimers observed by Kravets et al. could break membranes in a similar way, but further work is needed to explore this possibility. That said, despite these open questions, the work of Kravets et al. could ultimately lead to the development of novel anti-parasitic therapies.
